# Modulation of Corticospinal Excitability Depends on the Pattern of Mechanical Tactile Stimulation

**DOI:** 10.1155/2018/5383514

**Published:** 2018-04-03

**Authors:** Sho Kojima, Hideaki Onishi, Shota Miyaguchi, Shinichi Kotan, Ryoki Sasaki, Masaki Nakagawa, Hikari Kirimoto, Hiroyuki Tamaki

**Affiliations:** ^1^Institute for Human Movement and Medical Sciences, Niigata University of Health and Welfare, Niigata City, Japan; ^2^Department of Physical Therapy, Niigata University of Health and Welfare, Niigata City, Japan; ^3^Department of Rehabilitation, Tohoku University Hospital, Sendai City, Japan; ^4^Department of Rehabilitation, Kanto Central Hospital, Setagaya-Ku, Japan; ^5^Department of Sensorimotor Neuroscience, Graduate School of Biomedical & Health Sciences, Hiroshima University, Hiroshima City, Japan

## Abstract

We investigated the effects of different patterns of mechanical tactile stimulation (MS) on corticospinal excitability by measuring the motor-evoked potential (MEP). This was a single-blind study that included nineteen healthy subjects. MS was applied for 20 min to the right index finger. MS intervention was defined as simple, lateral, rubbing, vertical, or random. Simple intervention stimulated the entire finger pad at the same time. Lateral intervention stimulated with moving between left and right on the finger pad. Rubbing intervention stimulated with moving the stimulus probe, fixed by protrusion pins. Vertical intervention stimulated with moving in the forward and backward directions on the finger pad. Random intervention stimulated to finger pad with either row protrudes. MEPs were measured in the first dorsal interosseous muscle to transcranial magnetic stimulation of the left motor cortex before, immediately after, and 5–20 min after intervention. Following simple intervention, MEP amplitudes were significantly smaller than preintervention, indicating depression of corticospinal excitability. Following lateral, rubbing, and vertical intervention, MEP amplitudes were significantly larger than preintervention, indicating facilitation of corticospinal excitability. The modulation of corticospinal excitability depends on MS patterns. These results contribute to knowledge regarding the use of MS as a neurorehabilitation tool to neurological disorder.

## 1. Introduction

Somatosensory input is a widely used intervention in rehabilitation of neurological disorders such as strokes [1, 2]. This is because studies suggest that sustained somatosensory inputs such as electrical stimulation [3–10], vibration [11, 12], whole-hand water flow [13], and tactile stimulation [14] modulate cortical and corticospinal excitability. For example, motor-evoked potential (MEP), which indicates corticospinal excitability, was increased for 15 min following electrical stimulation of ulnar nerve for 2 h [3]. Additionally, vibration stimulated for 30 min on the palm of the hand increased corticospinal excitability for 2 h [11]. Corticospinal excitability modulation was induced by a change in the cortical excitability because F waves, which indicate spinal excitability, were unaffected by somatosensory input intervention [10, 11, 15]. Furthermore, short-interval intracortical inhibition (SICI) and intracortical facilitation (ICF) using paired pulse-transcranial magnetic stimulation (TMS) were changed by these interventions [7, 11, 13]. This suggests that the effects of somatosensory input reflect the modulation of facilitatory or inhibitory cortical circuits [7, 10, 11, 13].

Moreover, the effects of somatosensory input on corticospinal excitability depend on stimulus intensity, frequency, duration, and duty cycle (stimulus on/off cycle), thereby resulting in increased or decreased excitability [3–7, 10, 11, 16]. Chipchase et al. [16] compared the effect of 30 min of electrical stimulation on sensory and motor threshold intensities. Corticospinal excitability was increased by motor threshold stimulation and decreased by sensory threshold stimulation. Moreover, 20 min of mechanical vibration at 25 Hz increased corticospinal excitability, whereas the same at 10 Hz showed no effect [11]. These findings suggest that modulation of corticospinal excitability depends on somatosensory input conditions.

Mechanical tactile stimulation (MS) is a somatosensory input tool and was reported to alter neurophysiological and sensory skills [17–25]. A MS of 1 Hz for 3 h lowered the two-point discrimination threshold and shifted the localization of the N20 dipole of the index finger [19, 20]. The effects of MS depended on stimulus duration, frequency, and area [17, 19, 20, 23]. Previous studies have demonstrated changes in activity of the primary somatosensory cortex (S1) and sensory perception of stimulated area; however, little is known about how the activity of the primary motor cortex (M1) and motor skills is changed. Previously, it was reported that changes in S1 excitability modulated M1 excitability [26, 27]; therefore, we predict that MS modulates S1 activity to influence M1 excitability and hypothesize that sustained MS intervention modulates corticospinal excitability via S1 excitability.

A previous study using tactile stimulation demonstrated that cortical activity varied depending on the tactile stimulation pattern. Functional magnetic resonance imaging (fMRI) analysis reported that cortical activity depends on the MS pattern and demonstrated that S1 was activated by simple and complex MS, whereas M1 was only activated by complex stimulation [28]. Additionally, it was reported that the activities of the secondary somatosensory, premotor, and posterior parietal cortices were induced by complex and not simple stimulation [29–31]. Based on these studies, we hypothesized that corticospinal excitability is modulated by MS intervention and that modulation of corticospinal excitability depends on MS intervention patterns.

Here, we used five MS intervention patterns (simple, lateral complex, rubbing, vertical complex, and random complex). In experiment 1, we set simple, lateral complex, and rubbing interventions based on a previous study [28], to investigate the effect of simple or complex MS intervention on corticospinal excitability. Moreover, in experiment 2, we set vertical and random complex interventions to investigate the effects of the directionality of complex MS intervention on intervention effects. This study aimed to investigate the effects of MS intervention on corticospinal excitability and determine whether these depend on MS intervention patterns.

## 2. Methods

### 2.1. Participants

Overall, 19 healthy volunteers [age, 20–30 years; mean ± standard division (SD), 23.9 ± 2.5 years; 13 men; 6 women] participated in this study (experiment 1: 14 subjects; experiment 2: 12 subjects, including 11 of the same subjects as in experiment 1). None of the participants engaged in drug use or used medication that affected their central nervous system. All participants provided written informed consent. This study was approved by the ethics committee of Niigata University of Health and Welfare and was conducted in accordance with the Declaration of Helsinki.

### 2.2. MEP Measurement

MEPs were recorded from the right fist dorsal interosseous (FDI) muscle using a silver/silver chloride electrode in a belly–tendon montage. Electromyogram signals were amplified 100x (A-DL-720-140 amplifier; 4 Assist, Tokyo, Japan), digitized at 10 kHz using an A/D converter (Power Lab 8/30; AD instruments, Colorado Springs, CO, USA), and analyzed using Lab Chart 7 (AD instrument).

We used monophasic pulse TMS to elicit MEP. TMS was delivered by a figure-eight-shaped coil (95 mm diameter) connected to a Magstim 200 square (Magstim, Dyfed, UK). The coil was held with the handle pointing backwards and laterally at ~45° to the sagittal plane. The optimal spot for eliciting MEPs was carefully determined in each participant and was defined as the point where the TMS consistently evoked a large MEP from the right FDI. The optimal coil position was marked on a cap worn by the subject. Moreover, the position and orientation of the coil was monitored throughout the experiment by MRI using the Visor2 TMS Neuronavigation System (eemagine Medical Imaging Solutions GmbH, Berlin, Germany). The optimal spot of the FDI muscle was recorded and the coil was manually held in place to maintain position. T1-weighted MRI was performed using a 1.5-T system before the experiment (Signa HD, GE Healthcare, Milwaukee, WI, USA). The TMS intensity was defined as the lowest stimulus intensity that induced an MEP with ~1 mV peak-to-peak amplitude in the relaxed right FDI [32–34].

### 2.3. Intervention of MS

**Figure 1:**
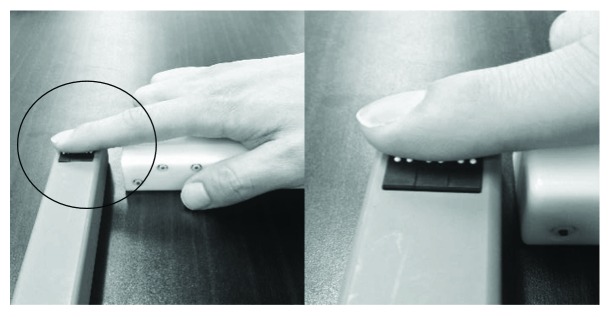
The settings of mechanical tactile stimulation. The mechanical tactile stimulator comprised 24 tiny plastic pins driven by piezoelectric actuators. A mechanical tactile stimulation was applied to the tip of the right index finger.

The mechanical tactile stimulator consisted of 24 tiny plastic pins driven by piezoelectric actuators (TI-1101; KGS, Saitama, Japan). The measurements for each pin were as follows: 1.3 mm diameter; height of the protrusion 0.8 mm with a pushing force of 0.031–0.12 N/pin [35, 36]. The distance between pins was set at 2.4 mm. An MS with 50 ms of protruding duration was applied to the tip of the right index finger (Figure 1). MS was applied for 20 min (stim on/stim off, 1 s/5 s) under the five following conditions: simple, lateral complex, rubbing, vertical complex, and random complex interventions. These interventions were classified into experiment 1 (simple, lateral complex, and rubbing) and experiment 2 (vertical complex and random complex).

**Figure 2:**
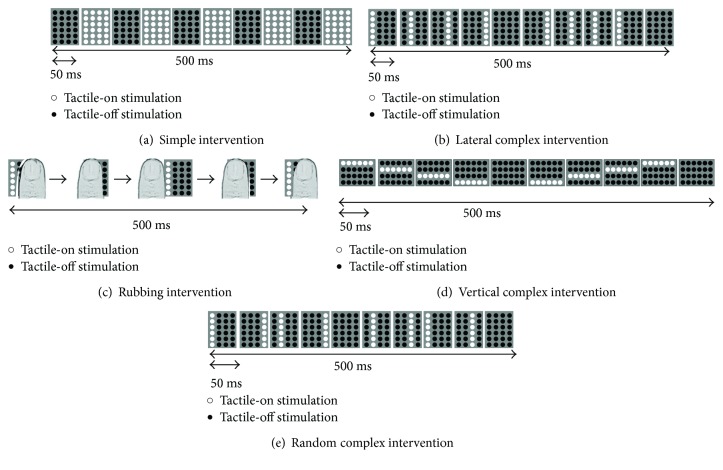
The condition of intervention (black dot: tactile-off, white dot: tactile-on). Five interventions (a–e) were applied for 20 min (stim on/stim off: 1 s/5 s).

Figure 2 shows the patterns of tactile intervention. Simple intervention stimulated the index finger concurrently with 24 pins that were installed in the finger pad (Figure 2(a)). Lateral complex intervention was stimulated by moving the row of six pins between the left and right side on the finger pad (Figure 2(b)). Rubbing intervention was stimulated by moving the stimulus probe, which is fixed by the protrusion pins and is controlled by machine (Figure 2(c)). Vertical complex intervention was stimulated by moving the row of pins forward and backward on the finger pad (Figure 2(d)). Random complex intervention stimulated the finger pad with moving either row of pins on the left and right (Figure 2(e)). The lateral complex, rubbing, and vertical complex interventions were set as moving two reciprocates on the finger pad in 1 s.

### 2.4. Study Design

**Figure 3:**
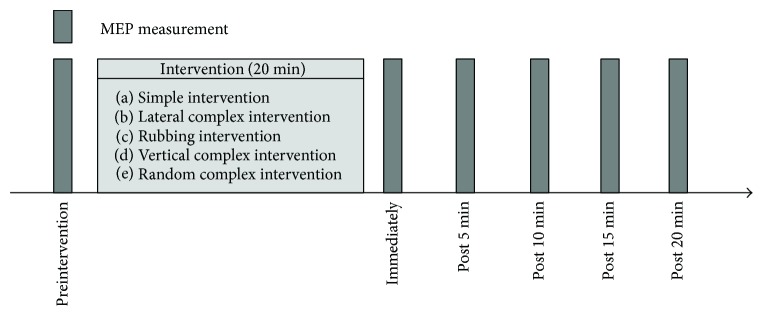
Experimental protocol. Motor-evoked potential as a measure of corticospinal excitability was measured before the intervention (preintervention), immediately after intervention (immediately), and 5 (post 5 min), 10 (post 10 min), 15 (post 15 min), and 20 min after the intervention (post 20 min). Transcranial magnetic stimulation was delivered in 15 trials at 0.2 Hz. The interventions performed in a repeated measurement design using a randomized order, with an interval of at least 1 week between each condition.

Participants were seated comfortably in a chair at rest with the forearm pronated. In all conditions, we monitored the lack of contraction of FDI during the intervention of MS and MEP measurement. During MEP measurement and intervention, participants were asked to continue looking at the front target to direct attention away from the right hand. MEPs were measured before the intervention (preintervention), immediately after intervention (immediately), 5 min after the intervention (post 5 min), 10 min after the intervention (post 10 min), 15 min after the intervention (post 15 min), and 20 min after the intervention (post 20 min); TMS pulses were delivered in 15 trials at 0.2 Hz (Figure 3). The interventions performed in a repeated measurement design using a randomized order, with an interval of at least 1 week between each condition.

### 2.5. Data and Statistical Analysis

Mean MEP amplitudes were calculated from the peak-to-peak amplitudes of 13 of the 15 trials, with elimination of the largest and the smallest values. Statistical analyses were carried out using SPSS statistics 21 software (IBM SPSS, Armonk, New York, USA). The TMS intensities were statistically analyzed by one-way repeated measures analysis of variance (ANOVA) for experiment 1 (intervention: simple, lateral complex, and rubbing) and paired *t*-test for experiment 2. The mean MEP amplitudes were statistically analyzed by two-way repeated measures ANOVA [intervention (experiment 1: simple, lateral complex, and rubbing; experiment 2: vertical complex and random complex) × time (preintervention, immediately, post 5 min, post 10 min, post 15 min, and post 20 min)], and we calculated the effect size of the ANOVA using partial eta-squared (partial *η*^2^). Post hoc analyses were performed using Dunnett's tests to compare each pre- and post-MS intervention. Moreover, post hoc analyses were conducted using Bonferroni's methods (experiment 1) and a paired *t*-test (experiment 2) to compare the effects of each intervention. Statistical significance was set at a *P* value of <0.05.

## 3. Results

In experiment 1, the intensity of TMS (mean ± SD) was 56.7 ± 6.7% maximum stimulator output (MSO) for the simple intervention, 59.1 ± 7.0% MSO for the lateral complex intervention, and 57.3 ± 5.8% MSO for the rubbing intervention. One-way repeated measures ANOVA revealed no significant difference in MSO between interventions [*F*(2,26) = 2.912, *P* = 0.072]. In experiment 2, the intensity of TMS was 56.2 ± 8.5% MSO for the vertical complex intervention and 56.5 ± 9.1% MSO for the random complex intervention. Paired *t*-test revealed no significant difference in MSO between interventions (*P* = 0.813).

### 3.1. Experiment 1

Two-way repeated measures ANOVA revealed a significant effect of intervention [*F*(2,26) = 34.59, *P* < 0.001, partial *η*^2^ = 0.231] and time [*F*(5,65) = 4.62, *P* = 0.001, partial *η*^2^ = 0.046] on MEP amplitudes. In addition, there was a significant interaction between intervention and time [*F*(10,130) = 7.30, *P* < 0.001, partial *η^2^* = 0.147].

**Figure 4:**
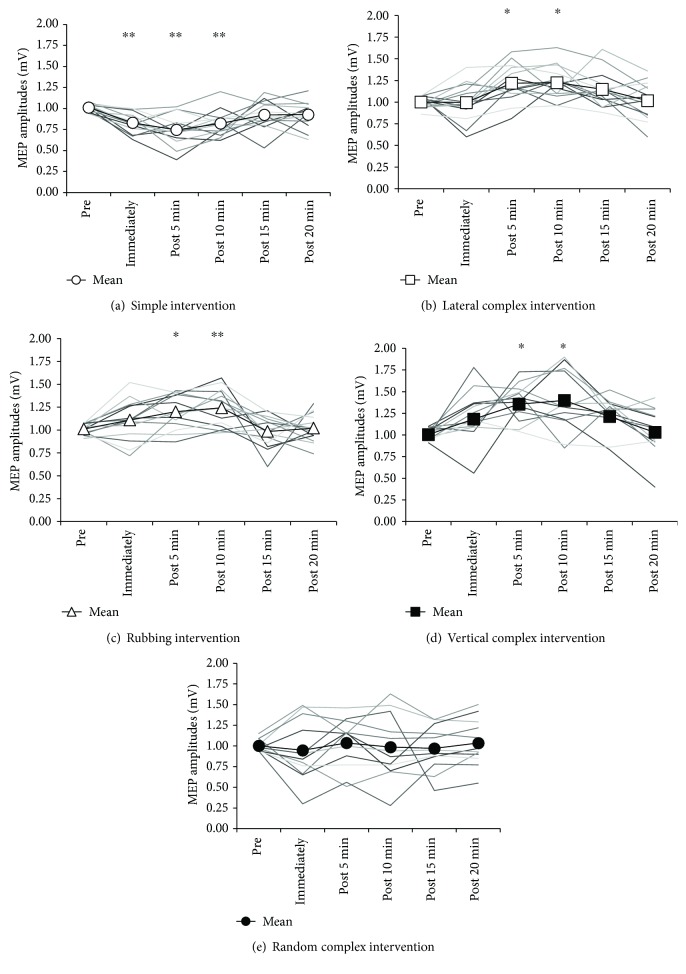
The motor-evoked potential (MEP) amplitude of each subject and the mean before and after interventions (black line: mean data, gray line: each subject data). (a) The mean MEP amplitudes were significantly smaller immediately 5 min and 10 min after simple intervention than they were preintervention (immediately; *P* = 0.006, post 5 min; *P* < 0.001, post 10 min; *P* = 0.004). (b) The mean MEP amplitudes were significantly larger 5 min and 10 min after lateral complex intervention than they were preintervention (post 5 min; *P* = 0.016, post 10 min; *P* = 0.013). (c) The mean MEP amplitudes were significantly larger 5 min and 10 min after rubbing intervention than they were preintervention (post 5 min; *P* = 0.024, post 10 min; *P* = 0.004). (d) The mean MEP amplitudes were significantly larger 5 and 10 min after vertical complex intervention than they were preintervention (post 5 min; *P* = 0.024, post 10 min; *P* = 0.003). (e) The mean MEP amplitudes were not significantly different between prerandom complex intervention and postintervention. (^∗^; *P* < 0.05, ^∗∗^; *P* < 0.01).

In the simple intervention, the mean MEP amplitude [mean ± standard error of the mean (SEM)] was 1.01 ± 0.01 mV (preintervention), 0.83 ± 0.03 mV (immediately), 0.75 ± 0.05 mV (post 5 min), 0.82 ± 0.04 mV (post 10 min), 0.92 ± 0.05 mV (post 15 min), and 0.93 ± 0.04 mV (post 20 min). Post hoc analyses revealed that MEP amplitudes were significantly smaller immediately, 5 min, and 10 min after intervention than they were at preintervention (immediately; *P* = 0.006, post 5 min; *P* < 0.001, post 10 min; *P* = 0.004). There was no significant difference in MEP amplitudes between preintervention and 15 or 20 min after intervention (*P* > 0.05) (Figure 4(a)).

In the lateral complex intervention, the mean MEP amplitude (mean ± SEM) was 1.00 ± 0.02 mV (preintervention), 1.00 ± 0.06 mV (immediately), 1.22 ± 0.06 mV (post 5 min), 1.23 ± 0.05 mV (post 10 min), 1.15 ± 0.06 mV (post 15 min), and 1.02 ± 0.06 mV (post 20 min). Post hoc analyses showed that MEP amplitudes were significantly larger 5 min and 10 min after intervention than they were preintervention (post 5 min; *P* = 0.016, post 10 min; *P* = 0.013). There was no significant difference in MEP amplitude between preintervention and immediately, 15 min, or 20 min after intervention (*P* > 0.05) (Figure 4(b)).

In the rubbing intervention, the mean MEP amplitude (mean ± SEM) was 1.01 ± 0.02 mV (preintervention), 1.11 ± 0.06 mV (immediately), 1.20 ± 0.05 mV (post 5 min), 1.24 ± 0.06 mV (post 10 min), 0.99 ± 0.05 mV (post 15 min), and 1.02 ± 0.04 mV (post 20 min). Post hoc analyses revealed that MEP amplitudes were significantly larger 5 min and 10 min after intervention than they were preintervention (post 5 min; *P* = 0.024, post 10 min; *P* = 0.004). There was no significant difference in MEP amplitude between preintervention and immediately, 15 min, or 20 min after intervention (*P* > 0.05) (Figure 4(c)).

Comparing all the interventions, the mean MEP amplitude immediately measured after rubbing intervention was significantly larger than that of simple intervention (*P* = 0.001), that of post 5 min and post 10 min after lateral complex and rubbing interventions was significantly larger than that of simple intervention (*P* < 0.001), and that of post 15 min after lateral complex intervention was significantly larger than that of simple intervention (*P* = 0.007). No significant difference was observed in MEP amplitude at other time points (*P* > 0.05).

### 3.2. Experiment 2

Two-way repeated measures ANOVA revealed the significant effect of intervention [*F*(1,11) = 6.98, *P* = 0.023, partial *η^2^* = 0.388] and time [*F*(5,55) = 2.98, *P* = 0.019, partial *η^2^* = 0.213] on MEP amplitudes. In addition, there was a significant interaction between intervention and time [*F*(5,55) = 4.01, *P* = 0.004, partial *η^2^* = 0.267].

In vertical complex intervention, the mean MEP amplitude (mean ± SEM) was 1.02 ± 0.02 mV (preintervention), 1.23 ± 0.09 mV (immediately), 1.38 ± 0.06 mV (post 5 min), 1.39 ± 0.10 mV (post 10 min), 1.24 ± 0.06 mV (post 15 min), and 1.06 ± 0.08 mV (post 20 min). Post hoc analyses revealed that MEP amplitudes were significantly larger 5 and 10 min after intervention than they were preintervention (post 5 min; *P* = 0.024, post 10 min; *P* = 0.003). There was no significant difference in MEP amplitude between preintervention and immediately, 15 min, or 20 min after intervention (*P* > 0.05) (Figure 4(d)).

In the random complex intervention, the mean MEP amplitude (mean ± SEM) was 1.00 ± 0.02 mV (preintervention), 0.95 ± 0.11 mV (immediately), 1.04 ± 0.09 mV (post 5 min), 0.99 ± 0.11 mV (post 10 min), 0.97 ± 0.08 mV (post 15 min), and 1.04 ± 0.08 mV (post 20 min). There was no significant difference at each MEP amplitude (*P* > 0.05, Figure 4(e)).

Comparing all interventions, the mean MEP amplitude of post 5 min, post 10 min, and post 15 min after vertical complex intervention was significantly larger than that of random complex intervention (*P* < 0.05). No significant difference was observed in MEP amplitude at other time points (*P* > 0.05).

## 4. Discussion

In this study, we investigated the effects of MS on MEP amplitudes to clarify the modulation of corticospinal excitability using different patterns of mechanical tactile stimulation. In experiment 1, simple intervention decreased the MEP amplitude immediately, 5 min, and 10 min after MS intervention, indicating the depression of corticospinal excitability. In contrast, lateral complex and rubbing intervention increased MEP amplitudes 5 min and 10 min after MS intervention, indicating the facilitation of corticospinal excitability. Moreover, in experiment 2, vertical complex intervention increased MEP amplitudes 5 and 10 min after MS, indicating facilitation of corticospinal excitability. In contrast, random complex intervention did not result in a significant difference at each MEP amplitude. These results suggested that MS intervention modulates corticospinal excitability and that this depends on the pattern of MS.

### 4.1. Inhibitory Effects of Mechanical Tactile Stimulation

In this study, MS by simple intervention decreased the MEP amplitude immediately, 5 min, and 10 min after intervention. Previous studies have reported that the electrical stimulation of the digital or median nerve for 30 min decreases the MEP amplitude for 10–30 min [5–7]. Moreover, stimulus intensities below the motor threshold either suppressed or did not affect the MEP amplitude [5–7, 15]. The change of MEP amplitudes by electrical stimulation has been suggested to depend on the intensity of electrical stimulation [16]. In addition, Onishi et al. [36] recorded the cortical activity (somatosensory-evoked magnetic field) following MS by magnetoencephalography (MEG) and demonstrated that the responses of S1 by MS were similar to that elicited by electrical stimulation. Therefore, we assume that the effects of simple intervention in this study are the same as the effects of electrical stimulation using a weaker intensity.

It was previously shown that MS intervention changed the two-point discrimination, the area, and strength of S1 activity. Moreover, these effects have been reported to depend on the duration time, stimulus frequency, and stimulus area [17, 20, 23]. Godde et al. [17] tested the efficiency of MS by comparing 6 h, 2 h, and 0.5 h stimulation and showed that MS intervention for 2 h modulated two-point discrimination. Electroencephalogram (EEG) or fMRI analyses have been used to investigate the neurophysiological changes induced by simple MS for 3 h; this intervention increased the area and strength of S1 activity [19, 20]. In addition, a significant correlation was observed between perceptual discrimination and changes in S1 activity [20]. Ragert et al. [23] reported that a brief 20 min period of intermittent high-frequency (20 Hz) simple MS decreased the two-point discrimination thresholds of finger stimulation. These findings suggest that the simple MS increases S1 activity. On the other hand, M1 excitability was increased at rest following cooling of S1, and the decreased excitation of pyramidal cells in S1 may alter M1 activity through long-range connections from S1 [26, 27]. Furthermore, Schabrun et al. [9] showed that the electrical stimulation of sensory thresholds increased the amplitudes of somatosensory-evoked potentials with decreasing MEP amplitudes, suggesting a correlation between S1 and M1 excitability. Therefore, the suppression of corticospinal excitability observed in this study might reflect the suppressed excitability of M1 by increasing S1 excitability in response to simple intervention. To examine these possibilities, we intend to investigate the effects of simple MS intervention on S1 excitability using an EEG or MEG that can measure S1 activity. One limitation of experiment 1 was that although the stimulus width was the same in each intervention, the total number of pins varied between simple and complex (lateral and rubbing) interventions. Therefore, in future studies, we aim to evaluate the effects of the total number of pins on corticospinal excitability.

### 4.2. Facilitatory Effects of Mechanical Tactile Stimulation

The current study demonstrates that lateral complex, vertical complex, and rubbing interventions increased the MEP amplitude at 5 and 10 min after intervention, indicating the facilitation of corticospinal excitability. Lateral complex intervention caused stimulation by moving a row of 6 pins, whereas rubbing caused stimulation by moving the stimulus probe, which was fixed by protrusion pins and was mechanically controlled. Lateral complex and rubbing interventions had the same degree of stimulus width and a similar approach that the stimulus was moved from left to right. These moving complex interventions increased the corticospinal excitability after intervention. A previous study reported that the cortical activities of somatosensory input depend on the peripheral stimulation technique. Comparisons of simple and complex MS with lateral moving using fMRI have demonstrated that S1 was activated by simple and moving complex MS, whereas M1 was only activated by moving complex stimulation [28]. A previous study of tactile motion involving animals reported that part of a cortical neuron is highly sensitive to the direction of stimulus motion, and tactile moving speed may relate the strength of the response of cutaneous mechanoreceptive afferents [37]. Based on our results, we predict that the varied effects of simple versus moving complex intervention result from the activity of this neuron. Moreover, we investigated the corticospinal excitability facilitation factor by moving complex intervention in experiment 2. We observed that vertical complex intervention with direction in motion increased corticospinal excitability, whereas random complex intervention without direction in motion remained unchanged. The facilitation of corticospinal excitability was reported following water flow or vibration using a roller-like machine [11, 13]. Sato et al. [13] demonstrated that whole-hand water flow with single directionality increased corticospinal excitability, whereas whole-hand water immersion did not. As shown in the results of experiments 1 and 2, directionality complex intervention that stimulates via a single direction, such as lateral complex, vertical complex, and rubbing interventions, increased MEP amplitude, indicating increased corticospinal excitability. However, random complex intervention without directionality did not modulate MEP amplitude. The four complex interventions (lateral, vertical, rubbing, and random) were set with the same parameters as of stimulus area, total pin number, and protruding duration of the pin. The directionality of stimulus movement was included in the three complex intervention (lateral, vertical, and rubbing), but not in random complex intervention. Therefore, these results, in addition to those of previous studies, suggest that the facilitation of corticospinal excitability is induced by directionality complex intervention that stimulates with a single direction rather than simple or complex intervention without directionality.

Moreover, Christova et al. [11] reported that 20 min vibration increased MEP amplitudes without modulating F waves, which are associated with spinal excitability. This suggested that the effects of vibration are caused by changes in cortical excitability rather than spinal excitability. Sato et al. [13] reported that water flow affected SICI and ICF with increasing MEP amplitude. Based on previous studies, it is considered that the facilitation of MEP amplitude by tactile intervention in this study is caused by modulation of intracortical excitability. Furthermore, previous studies of tactile motion have observed that the activity of the secondary somatosensory, premotor, and posterior parietal cortices was clearly observed by complex stimulation and not than simple stimulation [29–31]. Because these areas all have functional connectivity with M1, it is possible that the MEP facilitated by directionality complex intervention is related to the activity of these areas. We plan to investigate the effects of MS intervention on intracortical and spinal excitability or cortico-cortical network in the future.

The MEP amplitude was significantly larger 5 min and 10 min after intervention than it was preintervention, whereas the MEP amplitude was not affected immediately after lateral complex, vertical complex, or rubbing MS. This indicated a delayed effect. We have no definite explanation for why observed a delayed effect. However, previous studies have reported delayed rather than immediate corticospinal excitability induced by noninvasive S1 stimulation [34, 38, 39]. MEP amplitudes were facilitated by modulated S1 excitability, and these effects were delayed rather than immediate.

## 5. Conclusions

We have demonstrated that simple intervention decreased corticospinal excitability, whereas lateral complex, rubbing, and vertical complex interventions increased corticospinal excitability, indicating that the modulation of corticospinal excitability depends on the pattern of MS. Moreover, random complex intervention without directionality did not modulate corticospinal excitability, indicating that the facilitation of corticospinal excitability is induced by directionality complex intervention that stimulates with a single direction. Current results contribute to knowledge regarding the use of MS as a neurorehabilitation tool to neurological disorder.
